# Barriers and Facilitators to Accessing Psychosocial Support Following Miscarriage: A Scoping Review Protocol

**DOI:** 10.1002/hsr2.72437

**Published:** 2026-04-27

**Authors:** Alice Hone, Einar Thorsteinsson, Joseph Turner, Sally Bristow, Henri Dohnt

**Affiliations:** ^1^ University of New England Armidale New South Wales Australia

**Keywords:** early pregnancy loss, miscarriage, perinatal mental health, psychosocial support

## Abstract

**Background and Aims:**

Miscarriage is the most common complication in early pregnancy and is associated with adverse mental health outcomes. Expectant parents want emotional support during this devastating life event, yet few receive the support they desire. Access to support may be affected by individual, societal, and systemic factors. This study aims to map international English literature about the barriers and facilitators to accessing psychosocial support. As miscarriage can have enduring psychological impacts, it is pertinent to uncover factors impacting access to psychosocial support, as this may reduce the psychological impact of miscarriage and promote wellbeing.

**Methods:**

Any peer‐reviewed English literature that explores barriers and facilitators to accessing psychosocial support in an adult population experiencing miscarriage will be considered for inclusion in the review, including perceptions from partners and health professionals. Literature that assesses other forms of pregnancy loss (e.g., ectopic pregnancy, molar pregnancy, termination of pregnancy, stillbirth, neonatal death) is out of scope. To ensure methodological rigour and transparency, the planned scoping review will be conducted according to the Preferred Reporting Items for Systematic Reviews and Meta‐analysis extension for Scoping Reviews (PRISMA‐ScR) guidelines. A systematic search of PubMed/MEDLINE, PsycINFO, Web of Science, and CINAHL will be conducted to identify relevant literature.

**Results:**

The final scoping review will present the results of the search, study inclusion, and data analysis. Quantitative data will be presented in tabular format, stratified by geographical location and definition of miscarriage. Qualitative data will be coded and synthesized using descriptive qualitative content analysis. Barriers and facilitators will be presented visually using a socio‐ecological model framework whilst recommendations to minimize barriers and enhance facilitators will be tabulated.

**Conclusion:**

The planned review is the first of its kind to identify barriers and facilitators to accessing psychosocial support following miscarriage. Outcomes have the potential to advance understanding about access to psychosocial support, which could enhance miscarriage care and alter the psychological trajectory of people impacted by miscarriage.

## 1 Introduction

Miscarriage is the most common complication in early pregnancy, with an estimated 23 million miscarriages occurring worldwide each year [1]. Whilst definitions of miscarriage and recurrent miscarriage (or recurrent pregnancy loss) vary across countries and organizations, miscarriage is generally defined as the loss of a pregnancy prior to viability [1], whilst recurrent miscarriage has been defined as the loss of either two or more [2] or three or more pregnancies [3], that may or may not be consecutive, depending on the definition utilized.

Irrespective of the number of miscarriages an individual experiences, miscarriage can be a devastating life event and have profound psychological impacts. People affected by miscarriage frequently report that their loss has been minimized and their emotional experience invalidated by both health professionals and their social networks [4, 5, 6]. This can lead to disenfranchised grief, guilt, anger, self‐blame and may ultimately alter help‐seeking behavior [5, 6]. These experiences can contribute, compound, and prolong the psychological sequelae of miscarriage, which includes grief, severe depression and anxiety, trauma, and suicide [1, 7]. Partners can also experience short‐ and long‐term emotional distress [8, 9]. Women who have experienced miscarriage may have heightened anxiety, fear, and worry in subsequent pregnancies which may impact infant‐mother attachment and child‐rearing [5, 10].

Despite the serious psychological impacts of miscarriage, and implications for future pregnancies and child‐rearing, a lack of emotional support has consistently been reported across continents [8, 11]. Psychosocial support has been defined as the provision of psychological and social resources to an individual to assist them to cope with stressors and enhance wellbeing [12]. Psychosocial support may be formal and provided by professional service providers (e.g., therapists, doctors, obstetricians, midwives, peer support services or support groups) or informal and provided by those who have a social relationship with the individual (e.g., family, friends, colleagues, communities) [13]. It may take the form of practical, tangible, or emotional support [14]. Irrespective of the form, social support is most effective when it is aligned with an individual's needs and preferences, and is perceived by them as helpful [15].

Miscarriage has a higher prevalence than both stillbirth and neonatal loss combined [16, 17]. Despite this, there is a paucity of research into psychosocial support following miscarriage, in contrast to what is available for the other forms of perinatal loss [1]. Across continents, quantitative and qualitative research on miscarriage care has focused on the psychological impact of miscarriage, medical management of miscarriage, and experiences of care in the hospital system, rather than post‐miscarriage. This is a time when follow‐up care is lacking or focused exclusively on physical symptoms [18] and individuals are at heightened risk of developing mental health conditions [19, 20]. In addition, whilst social support is one of the most accessible forms of psychosocial support individuals can receive and has been shown to enhance wellbeing and aid recovery from miscarriage [21, 22, 23, 24], it has received less empirical attention. Furthermore, individual studies suggest that access to psychosocial care may be impacted at the individual, societal, and systemic level. The Social‐Ecological Model (SEM) provides a theoretical framework to assist in understanding peoples' experiences of miscarriage and factors which may influence peoples' access to psychosocial support. A comprehensive review to synthesize the barriers and facilitators to accessing psychosocial support is warranted to appropriately address this important, yet under‐researched, dimension of miscarriage care.

At the individual level, factors such as perception of the loss (i.e., loss of a baby or loss of pregnancy), perceived self‐efficacy [25], internalization of societal views, and coping mechanisms may impact access to support services. At the societal level, cultural norms and values have been shown to be relevant. Historically, miscarriage has been silenced in society and viewed as a taboo subject across cultures, is stigmatized and misunderstood [26]. In addition, people affected by miscarriage frequently report a lack of understanding from family, friends, and health care professionals which may impact access to support [23, 27]. This may act as a barrier to both asking and accessing psychosocial support from health care professionals and social networks. Systemic factors, such as health care provider factors, policy, and political factors, are also worthy of consideration [28]. Access to services may be impacted by a lack of government funding to support lower‐cost psychosocial support services, a lack of specialized services or trained professionals [27], lack of awareness from health professionals regarding the psychological impacts of miscarriage [29, 30, 31]. Policy and service constraints may also impact support, such as time and resource limitations, compassion fatigue [32], lack of clarity regarding role [33] and long waiting lists.

Understanding the barriers and facilitators to accessing psychosocial support for people impacted by miscarriage is essential to improve the quality of miscarriage care and reduce the psychological impact of miscarriage.

A preliminary search of MEDLINE, the Cochrane Database of Systematic Reviews, *JBI Evidence Synthesis*, FigShare, and Open Science Framework was conducted. No current or in‐progress scoping reviews or systematic reviews on the topic were identified.

The objective of this scoping review is to review and synthesize qualitative and quantitative evidence on the barriers and facilitators to accessing psychological or social support for people affected by miscarriage. It is pertinent to understand the factors that impact access to psychosocial support, as this has the potential to change the psychological trajectory of people impacted by miscarriage, their partners, and future family.

### 1.1 Review Question

The population, concept, context (PCC) framework [34] was used to inform the research questions (outlined in the following sections). The scoping review seeks to address the following research question: What are the barriers and facilitators to accessing psychosocial support from the perspectives of people affected by miscarriage and health professionals?

Additional aims of the present scoping review were to: (1) identify the rates and type of help‐seeking behavior, and perceived usefulness; (2) identify two independent lists of facilitators and barriers to accessing psychosocial support, categorized at the individual, societal, and systematic level.

### 1.2 Inclusion Criteria

#### 1.2.1 Population/Participants

Adults 18 years and older who have experienced a pregnancy loss prior to 24‐weeks gestation will be included in the review. There is marked variability in how miscarriage is defined across continents, thus this review takes an inclusive approach utilizing a 24‐week inclusion threshold to align with international standards. Experiences from people with recurrent miscarriage, late miscarriage, and their partners will also be included. Perspectives from medical or allied health professionals will be included. If studies do not report gestational age at time of loss, or if a mix of early (< 24 weeks) and late (> 24‐weeks) losses is reported, authors will be contacted to obtain this data. Studies will be excluded where losses occurred beyond 24‐weeks gestation. Consistent with previous literature, when data for gestational age at the time of loss cannot be isolated, studies will be excluded if 50% or more of the sample experienced a loss beyond 24‐weeks gestation. Individuals who have experienced other forms of pregnancy loss (e.g., ectopic pregnancy, molar pregnancy, termination of pregnancy, stillbirth or neonatal death) or abortion are outside the scope of this review.

#### 1.2.2 Concept

International literature that explores, describes or refers to individual, societal, systemic or contextual barriers or facilitators to accessing psychological or social support will be considered for inclusion in the review.

#### 1.2.3 Context

The review will focus on adults experiencing miscarriage, irrespective of the time or geographical location when the miscarriage occurred. Any setting with which participants attempted to access psychosocial support (e.g., primary care, secondary care, online support groups) will be considered. Data from non‐English languages were excluded due to resource limitations for achieving culturally responsive translation [35]. This exclusion mitigates the risk of losing subtle contextual meaning for accurately interpreting qualitative experiences of accessing psychosocial support following miscarriage [36].

### 1.3 Types of Sources

The present scoping review will consider any methodological or study design for inclusion, including peer‐reviewed quantitative, qualitative, mixed‐methods and reviews, irrespective of date of publication. Systematic or scoping reviews that meet the inclusion criteria will also be considered.

## 2 Methods

The proposed scoping review will be conducted according to the JBI methodology for scoping reviews [34], with results presented in accordance with the Preferred Reporting Items for Systematic Reviews and Meta‐analysis extension for Scoping Reviews (PRISMA‐ScR [37]) (see Supporting Information S1: PRISCMA‐ScR checklist). Details of this scoping review project are available in Open Science Framework (https://osf.io/qgxa7) (DOI 10.17605/OSF.IO/QGXA7). The protocol was registered in July 2025 prior to data extraction and any changes to the protocol will be identified in the final scoping review.

### 2.1 (Step 1) Search Strategy

In the development of the present scoping review, a research librarian was consulted to assist with the development of the search strategy. In addition, a preliminary search of PubMed/MEDLINE (NCBI) and CINAHL Complete (EBSCO) was carried out using MeSH and keyword combinations. The titles and abstracts of relevant articles were examined. Keywords and index terms identified from the articles were used to develop a full search strategy for PubMed/MEDLINE (see Table 1). The search strategy will be adapted for each included database and/or information source (see Supporting Information S2: full search strategies for all databases and Table 2). Databases to be searched in full include PubMed/MEDLINE (NCBI), PsycINFO (ProQuest), Web of Science (Clarivate), and CINAHL Complete (EBSCO). Only studies published in English will be included, irrespective of date of publication. Systematic or scoping reviews that meet criteria will also be included.

**Table 1:** PubMed/MEDLINE (NCBI) search strategy with limiters (English language, Humans, Abstracts) executed July 11, 2025.

Search	Search terms (MeSH and keyword)
1	(abortion, spontaneous[MeSH Terms])
2	“spontaneous abortion” OR miscarriage OR “early pregnancy loss” OR “perinatal loss” OR “first trimester loss”
3	1 or 2
4	(barrier* OR obstacle* OR disadvantage* OR difficult* OR problem OR challeng* OR inhibit* OR facilitat* OR assist* OR enabl*)
5	(social support[MeSH Terms]) OR (help‐seeking behavior[MeSH Terms])
6	“social support*” OR “psychosocial support*” OR “support group*” OR “help‐seek” or “help seek*” OR psycho*
7	5 OR 6
8	3 and 4 and 7

**Table 2:** CINHAL (EBSCO), PsycINFO (ProQuest), and Web of Science (Clarivate) search strategy with limiters (English language, Humans, Abstracts) executed July 11, 2025.

Search	Search terms (Keyword)
1	“spontaneous abortion” OR miscarriage OR “early pregnancy loss” OR “perinatal loss” OR “first trimester loss”
2	(barrier* OR obstacle* OR disadvantage* OR difficult* OR problem OR challeng* OR inhibit* OR facilitat* OR assist* OR enabl*)
3	(“social support*” OR “psychosocial support*” OR “support group*” OR “help‐seek” or “help seek*” OR psycho*)
4	1 AND 2 AND 3

### 2.2 (Step 2) Study Selection

Identified citations will be imported into EndNote referencing software [38] with duplicates identified and removed. Screening and selection of literature will be a multi‐stage process that will utilize Covidence Systematic Review Software [39]. The initial stage will involve piloting the charting procedure of titles and abstracts based on a priori eligibility criteria. In accordance with JBI recommendations [34], 25 source titles/abstracts will be randomly selected for independent review by the research team. Discrepancies will be communicated with the research team, a consensus reached, and the search strategy reviewed (if necessary) until a minimum of 85% agreement is reached. Partial independent dual screening will occur with author AH screening all titles and author DH reviewing a random sample of 20% of titles. If the 85% agreement threshold is not achieved, additional titles will be screened in 20% increments until a minimum of 85% consensus is reached. Agreement will be assessed via percentage agreement. Any disagreement will be resolved through discussion with the research team until a consensus has been reached. Sources that meet the inclusion criteria will be retrieved in full‐text and citation details imported into EndNote. Full‐text articles will be examined by AH and at least one other co‐author. Reasons for subsequent exclusion will be noted and reported in the scoping review. The results of the search will be reported in full and presented in a PRISMA‐ScR [37] flow diagram in the final scoping review (see Supporting Information S3: Sample PRISMA‐ScR diagram).

### 2.3 (Step 3) Data Extraction

A draft extraction form is provided (see Supporting Information S4: Data Extraction Template). Researchers modified the JBI data extraction template [34] to suit the needs of the present scoping review study. The extraction process will be piloted on a small selection of sources by AH and at least one other co‐author to assess appropriateness and reliability. Any modifications to the procedure will be noted in the final scoping procedure. Data will then be extracted from all included sources by AH using Covidence [39] and entered into a database. The data extracted will include specific details about the participants, concept, context, study methods and key findings relevant to the review question/s. Tabulated data for inclusion will be reviewed by a minimum of two authors. Any disagreements will be resolved through communication with a member of the research team. If required, authors of papers will be contacted to request missing or additional data.

### 2.4 (Step 4) Results/Data Analysis and Presentation

As scoping reviews are intended to be investigative, results of study characteristics will be presented descriptively and tabulated, as they relate to the population, context, and concept elements of this scoping review. Specifically, rates, types of help‐seeking behavior, and perceived usefulness of psychosocial support will be presented in tabular format, stratified by geographical location and definition of miscarriage. Where relevant, quantitative data will be integrated into the SEM and presented diagrammatically with qualitative data. Qualitative data will be coded and synthesized by two independent reviewers (A.H. & S.B.) using descriptive qualitative content analysis over a two‐stage process. An inductive codebook will be developed from the data which will then be iteratively refined and agreed upon by the two coders, with inter‐coder reliability assessed using Cohen's *κ* to ensure consistency. Once the inductive coding framework is established, the resulting codes and themes will be mapped deductively onto the SEM. This two‐stage approach ensures that the analysis remains grounded in the data while also enabling interpretation within an established theoretical framework. To address mixed or overlapping determinants, codes and themes will be assigned to multiple levels of the SEM, where appropriate. This multi‐level coding approach will capture the complexity of factors that span more than one level (e.g., influences that operate at both individual and system levels), ensuring that the analysis accurately reflects the interconnected nature of the determinants. Where appropriate, data will be stratified by key variables, including geographical location, World Bank Income category (high‐ vs. low‐/middle‐income countries), and miscarriage definition, to enable meaningful comparison across groups. This analysis will identify whether the identified psychosocial barriers and facilitators remain consistent across global settings or are sensitive to specific socioeconomic and cultural environments. Results will be presented visually using a SEM diagram. Recommendations to minimize barriers and enhance facilitators will be tabulated.

## 3 Conclusion

Miscarriage can have detrimental impacts on psychological well‐being. People impacted by miscarriage want psychosocial support; however, rarely receive the support they desire. Incorporating perspectives from people affected by miscarriage, their partners, and health professionals, will provide a comprehensive understanding of the barriers and facilitators to accessing psychosocial support following miscarriage.

A major limitation of this scoping review is the restriction to peer‐reviewed English‐language literature. Whilst this was intended to mitigate methodological risks of losing meaning through translation and interpreting qualitative information outside a culturally responsive lens [35, 36], the findings of this review do not capture the barriers or facilitators of accessing psychosocial support for people experiencing miscarriage across the continent and likely result in an under‐representation of culturally diverse perspectives. A discussion of the study's strengths, additional limitations, and directions for future research, including research gaps, will provide advancement for miscarriage care.

This has the potential to advance understanding about access to psychosocial support, which has the potential to change the psychological trajectory of people impacted by miscarriage, enhance miscarriage care, and reduce the economic burden of miscarriage.

## Author Contributions

**Alice Hone:** conceptualization, writing – original draft, methodology, writing – review and editing. **Einar Thorsteinsson, Joseph Turner, and Sally Bristow:** writing – review and editing, conceptualization, and supervision. **Henri Dohnt:** writing – review and editing.

## Ethics Statement

The authors have nothing to report.

## Conflicts of Interest

The authors declare no conflicts of interest.

## Positionality Statement

The primary investigator (A.H.) has lived experience of recurrent miscarriage. To enhance objectivity and reduce interpreter bias, all stages of the scoping review will involve input from multiple members of the research team, including study selection, data extraction, analysis and interpretation. Patient/public perspectives will be elicited from research that meets inclusion criteria and reported in the results.

## Transparency Statement

The lead author Alice Hone affirms that this manuscript is an honest, accurate, and transparent account of the study being reported; that no important aspects of the study have been omitted; and that any discrepancies from the study as planned (and, if relevant, registered) have been explained.

## Supporting information

Supporting File 1

Supporting File 2

Supporting File 3

Supporting File 4

## Data Availability

The protocol was submitted to the Open Science Framework (OSF) for registration. No datasets were used in this protocol. Relevant data from this study will be made available upon the completion of the planned scoping review.
