# Supplementary material for: Barriers and Facilitators to Accessing Psychosocial Support Following Miscarriage: A Scoping Review Protocol
Source: Health Sci Rep. 2026 Apr 27;9(5):e72437. doi: 10.1002/hsr2.72437 (PMC13121853; doi:10.1002/hsr2.72437)
Supplement: Supplementary file 2 — Supporting File 2 [file HSR2-9-e72437-s004.pdf]

Full Search Strategy for all Databases

Table 1 PubMed/MEDLINE (NCBI) search strategy with limiters (English language, Humans, Abstracts) completed executed July 11, 2025

Search	Search terms (MeSH and keyword)
1	(abortion, spontaneous[MeSH Terms])
2	"spontaneous abortion" OR miscarriage OR "early pregnancy loss" OR “perinatal loss” OR “first trimester loss”
3	1 or 2
4	(barrier* or OR obstacle* or OR disadvantage* or OR difficult* or OR problem or OR challeng* or OR inhibit* or OR facilit at* OR assist* OR enabl*)
5	(social support[MeSH Terms]) OR (help-seeking behavior[MeSH Terms])
6	“social support*” OR “psychosocial support*” OR “support group*” OR “help-seek” or “help seek*” OR psycho*
7	5 OR 6
8	3 AND 4 AND 7

Table 2 CINHAL (EBSCO), PsycINFO (ProQuest), and Web of Science (Clarivate) search strategy with limiters (English language, Humans, Abstracts) completed executed July 11 2025

Search	Search terms (keyword)
1	"spontaneous abortion" OR miscarriage OR "early pregnancy loss" OR “perinatal loss” OR “first trimester loss”
2	(barrier* OR obstacle* OR disadvantage* OR difficult* OR problem OR challeng* OR inhibit* OR facilitat* OR assist* OR enabl*)
3	(“social support*” OR “psychosocial support*” OR “support group*” OR “help-seek” or “help seek*” OR psycho*)
4	1 AND 2 AND 3
