# Supplementary material for: Barriers and Facilitators to Accessing Psychosocial Support Following Miscarriage: A Scoping Review Protocol
Source: Health Sci Rep. 2026 Apr 27;9(5):e72437. doi: 10.1002/hsr2.72437 (PMC13121853; doi:10.1002/hsr2.72437)
Supplement: Supplementary file 3 — Supporting File 3 [file HSR2-9-e72437-s001.pdf]

Planned PRISMA-ScR flow diagram

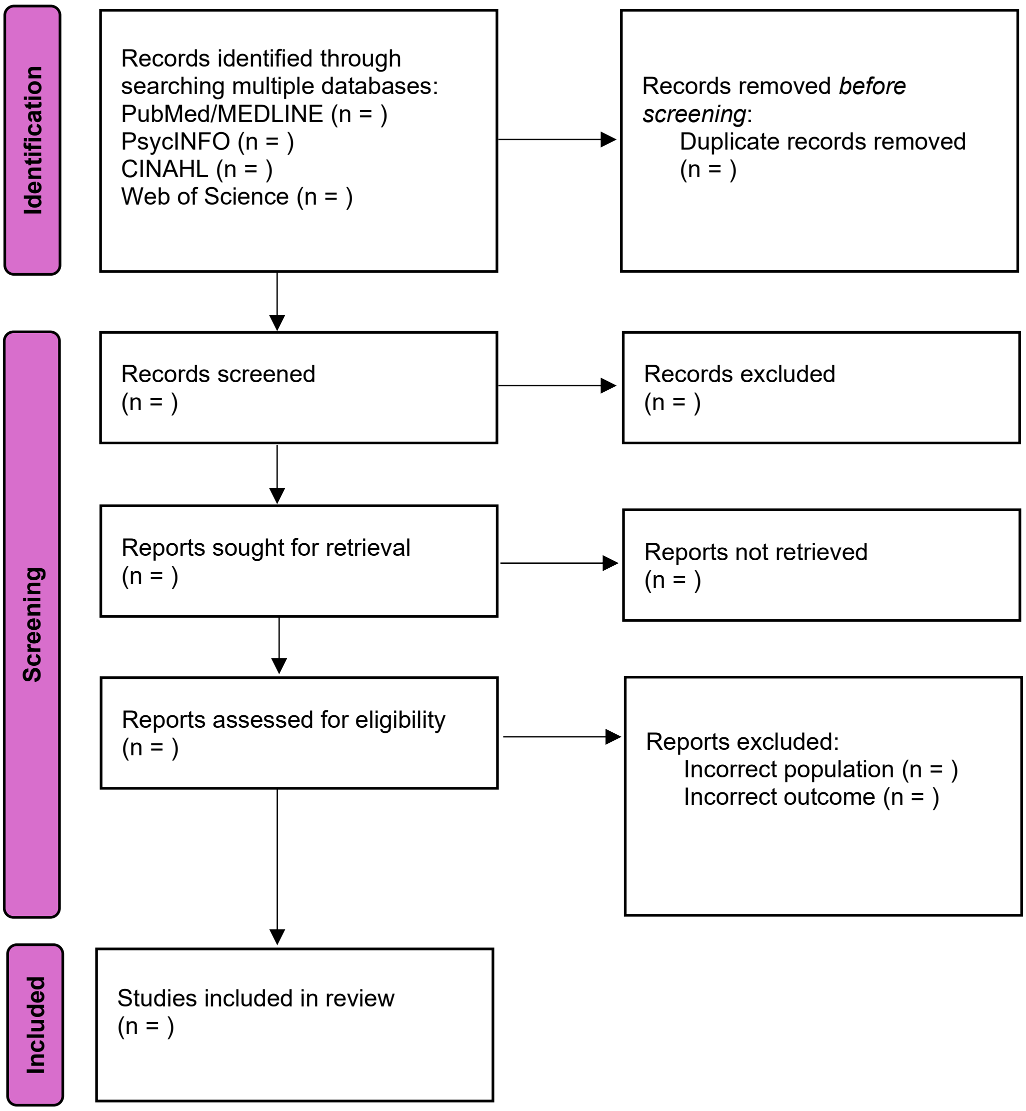

Adapted for the current scoping review protocol
Source: Page MJ, et al. BMJ 2021;372:n71. DOI: https://doi.org/10.1136/bmj.n71
Licensed under CC BY 4.0 (https://creativecommons.org/licenses/by/4.0/)
