# Supplementary material for: Barriers and Facilitators to Accessing Psychosocial Support Following Miscarriage: A Scoping Review Protocol
Source: Health Sci Rep. 2026 Apr 27;9(5):e72437. doi: 10.1002/hsr2.72437 (PMC13121853; doi:10.1002/hsr2.72437)
Supplement: Supplementary file 4 — Supporting File 4 [file HSR2-9-e72437-s003.pdf]

Data-Extraction Template

The below template was adapted from the JBI data extraction template (JBI evidence synthesis) and will be used for data extraction.

**Scoping Review Details**	
Scoping Review Title	
Review Objectives	
Review question/s	
**Inclusion/Exclusion Criteria**	
Population	
Concept	
Context	
Types of evidence source	
**Evidence Source Details**	
Author/Year	
Country	
Aims/Objective	
Study Design	
**Population & Participants**	
Population	
Sample Size	
Age (yrs) M & SD	
Gender	
Income (M & SD)	

Education (%)<br>Less than secondary schooling<br>Secondary schooling<br>College/Trade Certificate<br>Bachelor degree<br>Masters degree<br>Doctoral degree	
Marital status (%)<br>Single<br>Married<br>De-facto<br>Divorced<br>Separated<br>Widowed	
Identify as religious (%)	
Children before miscarriage (%)<br>0<br>1 or more	
Planned pregnancy (%)<br>Yes – wanted<br>Yes – unwanted<br>No – wanted<br>No - unwanted	
Duration to conceive (range in months/years)	
Duration since most recent miscarriage (range in months/years)	
Type of Miscarriage (M & SD)<br>Single<br>Recurrent	

Late<br><br>Missed<br><br>Spontaneous<br><br>Other	
# of Miscarriages (M & SD)	
Gestational age at loss (M & SD)	
% of sample seeking psychosocial support	
% of sample that<br>accessed psychosocial support	
**Concept**	
Conceptual framework	
Operational definition of psychosocial support	
Barriers (individual, social, cultural, health service, other)	
Facilitators (individual, social, cultural, health service, other)	
**Context**	
Setting (e.g., ED, EPAS, Community)	
Type of help-seeking behaviour<br>Formal: medical professional, health professional,<br>counsellor, psychologist, social worker, pastoral care, other<br><br>Informal: partner, family, friend, colleague, online social support group, other	

Mixed	
**Outcome**	
Perceived usefulness of help-seeking behaviour	
Study outcome	
